# A Case of Vogt-Koyanagi-Harada Disease and Retinal Peri-Phlebitis in a Patient With Presumed Ocular Tuberculosis

**DOI:** 10.7759/cureus.64200

**Published:** 2024-07-09

**Authors:** Muhammad Amjad, Aruba Zafar

**Affiliations:** 1 Vitreoretina, Al Shifa Trust Eye Hospital, Rawalpindi, PAK; 2 Ophthalmology, Al Shifa Trust Eye Hospital, Rawalpindi, PAK

**Keywords:** retinal peri-phlebitis, optical coherence tomography, tuberculous uveitis, vogt-koyanagi harada, exudative retinal detachment

## Abstract

A middle-aged hypertensive female presented with headaches, tinnitus, and blurred vision for two weeks. Clinical examination revealed mild vitritis and bilateral multifocal exudative detachments at the posterior pole, together with peripheral vascular cuffing and peri-phlebitis. Laboratory testing pointed towards isolated presumed intraocular tuberculosis (IOTB) as the probable cause. However, the patient strongly responded to high-dose intravenous and tapered oral corticosteroids, leading to complete resolution of detachments within 10 days of therapy initiation. Anti-tubercular therapy (ATT) was begun after one week of presentation, and no recurrence of symptoms was noted for the next 18 months. A case of Vogt-Koyanagi-Harada (VKH) disease-like presentation occurred after a probable previous subclinical episode(s) of presumed IOTB, resulting in sclerosed vessels in the retinal periphery.

## Introduction

Vogt-Koyanagi-Harada (VKH) disease is an autoimmune granulomatous multisystem inflammatory disease that can affect the eyes, meninges, and the integumentary and auditory systems. The exact mechanism of the T-lymphocyte-mediated process is unknown, but sensitization to melanocytic antigens after viral infections [1,2] or cutaneous injury has been identified as a possible trigger [3]. It usually presents as granulomatous pan-uveitis with exudative retinal detachments, optic disc hyperemia, and choroidal thickening. Here, we report a case of probable VKH disease but with the presence of some peculiar features that may be attributed to presumed intraocular tuberculosis (IOTB). Ocular tuberculosis (TB) is an infrequent manifestation of *Mycobacterium tuberculosis* (*M. tuberculosis*) infection that can affect various structures of the eye, including the choroid, retina, uvea, and optic nerve. Posterior uveitis is the most common clinical presentation of ocular TB, which may present with solitary or multiple choroidal tubercles and serpiginous-like choroiditis. Due to its nonspecific and diverse clinical presentation, ocular TB can be challenging to diagnose and often requires a high index of suspicion.

## Case presentation

A 55-year-old hypertensive, immunocompetent female presented with bilateral progressive painless visual blurring, persistent headaches, and tinnitus for two weeks. There was no history of ocular surgery or trauma. A review of systems was non-contributory, and she denied a history of trauma, ocular surgery, neurological symptoms, vaccination, weight loss, or travel. The patient's blood pressure was well controlled with medication, with no previous history of hypertensive urgency or emergency.

On examination, her Best Corrected Visual Acuity (BCVA) on the Snellen chart was 6/60 and 6/36 in the right (OD) and left (OS) eyes, respectively. Her pupils were regular, round, and reactive (RRR), and her extraocular movements were full in all directions. Intraocular pressures on Goldman Tonometry were 21 OD and 22 OS. On slit lamp examination, flare and +1 cells were noted in the anterior chamber (AC) in both eyes (OU), and trace cells and +1 haze were present in the posterior vitreous. Dilated fundoscopy revealed bilateral multifocal serous retinal detachments at the posterior pole, with markedly sclerosed vessels in the fundal periphery. No disc swelling, pigmentary changes, or lesions were otherwise seen. 

**Figure 1:**
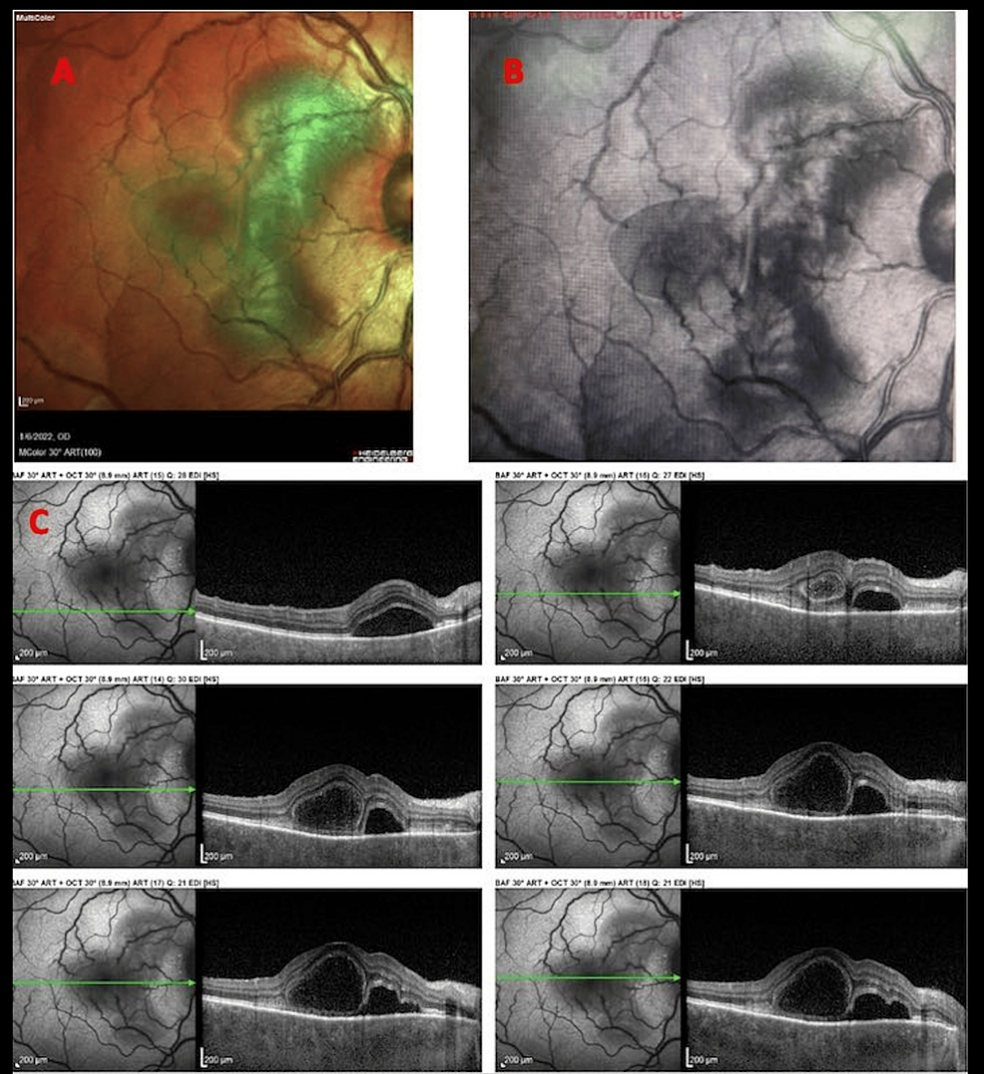
(A) and (B) Infrared images (OD) showing sharply demarcated multifocal detachments. (C) Optical coherence tomography raster scans (OD) through the macula showing multiple serous detachments, a bacillary layer detachment, and choroidal thickening.

**Figure 2:**
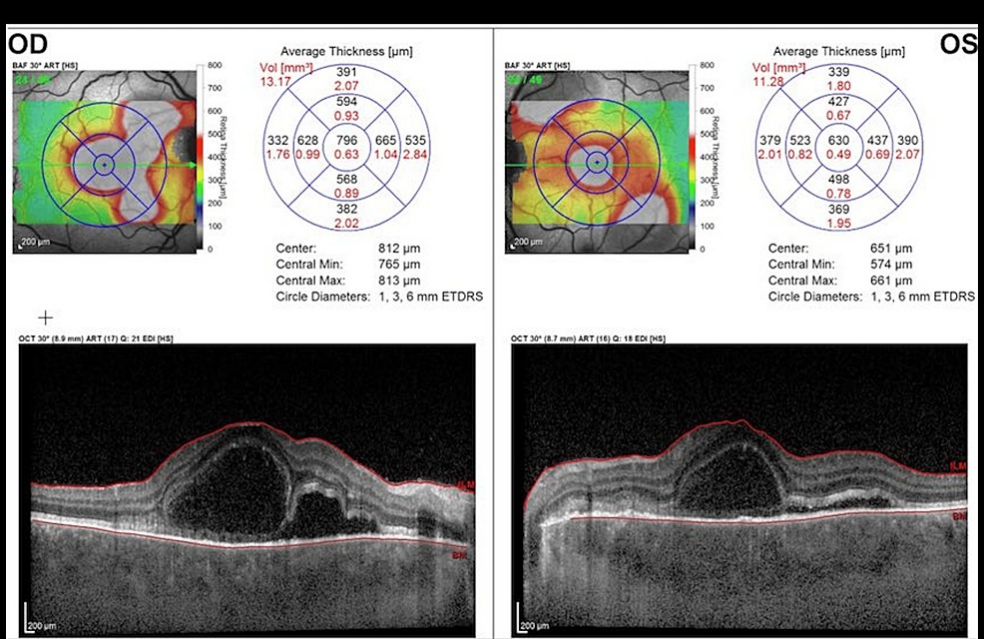
Optical coherence tomography (OCT) thickness map with bilateral multifocal serous detachments at the fovea.

**Figure 3:**
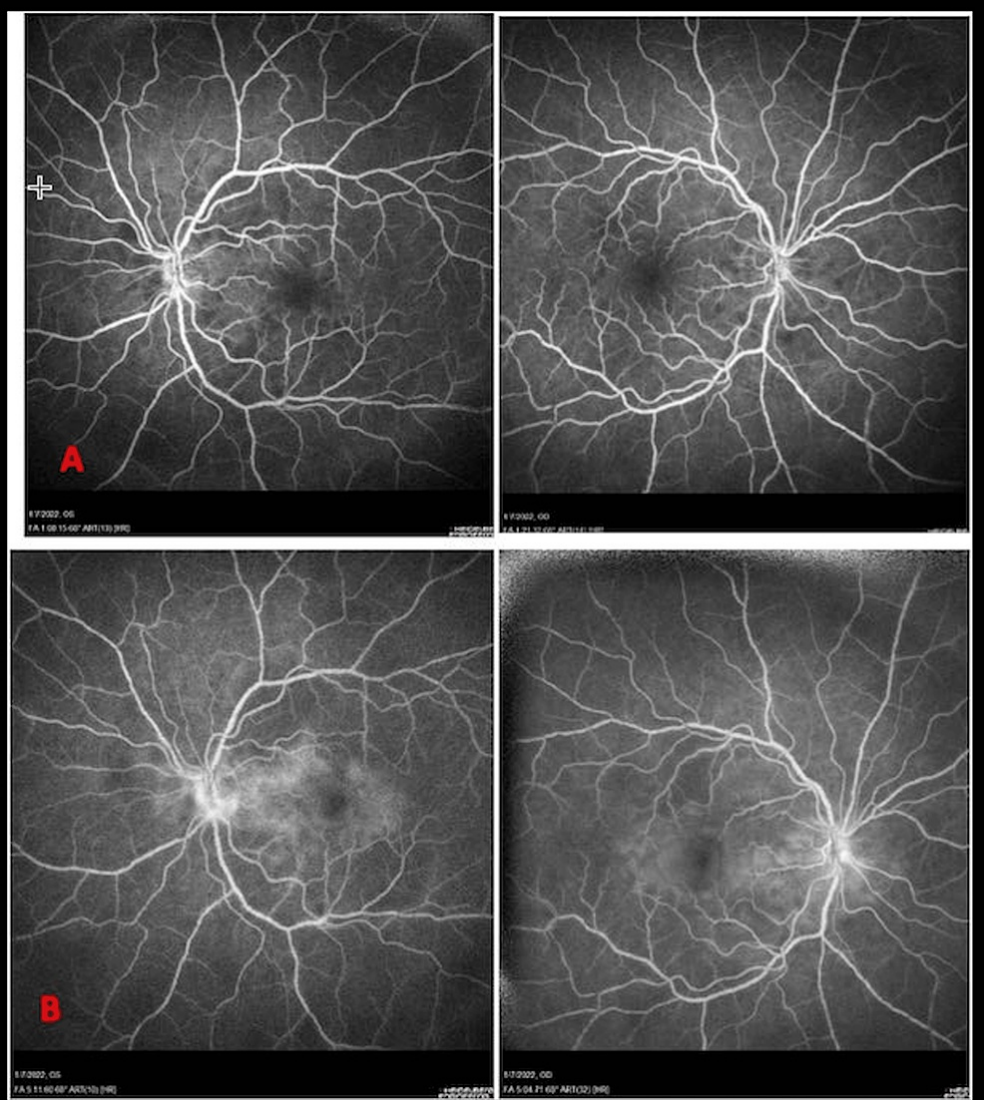
Fluorescein angiography OU showing (A) Bilateral patchy hypofluorescence in mid-phase and (B) Bilateral staining of optic discs and pooling of dye in areas of serous detachments in late phase.

On optical coherence tomography (OCT) (Figures 1-2), bilateral multifocal serous detachments were visible within the macular area, a bacillary layer detachment (BALAD) with an estimated increased choroidal thickness of 549 microns on enhanced depth imaging-OCT (EDI-OCT). Pathologic changes were grossly symmetric in both eyes. Fluorescein angiography was indicative of diffuse patchy hypopigmentation in the early films and late staining of the disc and leakage around the macula, coinciding with areas of serous detachments. No active vasculitic changes were noticeable (Figure 3). No evidence of posterior scleritis was noted on the ultrasound B-scan. 

Our initial differential diagnoses were broad, which included VKH disease, sarcoidosis, uveal effusion, infectious causes of uveitis like ocular TB and syphilis, and intraocular lymphoma. The patient was referred to a tertiary care center for administration of pulsed intravenous methylprednisolone 1 gm/day for three days, followed by oral prednisolone maintenance therapy (1 mg/kg/day for two weeks and then tapered). Meanwhile, laboratory investigations in consultation with a rheumatologist and infectious disease specialist were carried out. A complete blood count, erythrocyte sedimentation rate, C-reactive protein, syphilis screen, renal and liver function tests, viral hepatitis serology, urinalysis, and a complete rheumatologic profile, including anti-nuclear antibodies, cytoplasmic anti-neutrophil cytoplasmic antibodies (c-ANCA), perinuclear anti-neutrophil cytoplasmic antibodies (p-ANCA), anti-cardiolipin, anti-Smith (anti-SM) antibodies, etc., were all negative. Her serum hemoglobin electrophoresis was also normal. 

The only exception was a strongly positive interferon (IFN)-gamma assay for tuberculous antigen (QuantiFERON-TB Gold Plus; QIAGEN, Hilden, Germany) and a positive skin tuberculin test. The patient had a history of Bacillus Calmette-Guérin vaccination at birth. We obtained a chest X-ray, which was found to be within normal limits. High-resolution computed tomography (HRCT) imaging of her chest was not indicative of any bronchial or pulmonary pathology. Due to the strong possibility of further worsening with systemic steroids, the patient was counseled for treatment with a standard four-drug anti-tubercular therapy (ATT) regimen for presumed extrapulmonary TB as per WHO guidelines [4]. A polymerase chain reaction (PCR) or aqueous/vitreous sample was not conducted due to non-availability and the reported low yield of the organism in ocular samples in various studies [5]. We also could not conduct an indocyanine green angiography to detect the presence of choroidal tuberculomas.

Within a week of the commencement of IV steroid therapy and before administering oral anti-TB drugs, the patient showed a marked subjective and objective visual improvement, which on assessment was 6/9 OU. Her ACs were now quiet, and a repeat OCT showed nearly complete resolution of subretinal fluid along with a reduced choroidal thickness of approximately 374 microns. Her next follow-up was two weeks after beginning anti-TB therapy and showed no new signs or symptoms and no further deterioration. At 6- and 12-month follow-ups, her vision remained stable in both eyes (OU), and no recurrence was noted.

## Discussion

Tuberculous uveitis is one of the great mimickers of ocular inflammatory and infectious diseases and can have a wide range of clinical presentations. It can appear like many other entities, including VKH, syphilis, sarcoidosis, serpiginous choroiditis, and acute multifocal posterior placoid pigment epitheliopathy among others, and should be high on a clinician’s list of differentials, especially in endemic areas like Pakistan. Similarly, VKH disease features may be shared by multiple ocular entities. The early manifestation involves choroidal inflammatory signs with retinal exudative detachments, disc hyperemia, or vitritis, and late manifestations are otherwise non-specific with a pathognomonic finding of a “sunset glow” fundus. 

This case of bilateral multifocal serous detachments with BALAD and thickened choroid on OCT, which is classic for VKH disease [6], became a diagnostic dilemma due to the presence of retinal peri-phlebitis and an unexpectedly positive TB laboratory result. A robust clinical response to steroids and a reduction in choroidal thickness went strongly in favor of VKH. However, a positive IFN-gamma assay in a bilateral uveitis patient with peri-phlebitis does not fit the established criteria of VKH [7]. Therefore, ATT along with corticosteroids were administered not only because of a mixed picture but also the high prevalence of TB in our region.

A few cases with mixed features of VKH and TB have been reported in the literature, which posed challenges in diagnosis and management [8-10]. Such cases highlight the importance of keeping a low threshold of suspicion for intraocular TB in endemic areas because the long-term immunosuppression required to treat a diagnosed case of VKH can potentially worsen or lead to the dissemination of pre-existing TB. Multiple complications owing to relapses and chronicity are not unusual in VKH or TB. To date, there is no single test to definitively differentiate these two conditions. Human leukocyte antigen (HLA)-DR and DQ haplotyping to assess the genetic susceptibility for VKH and ocular fluid PCR to rule out tuberculous antigens may not be available or feasible in most endemic areas of the world. The diagnostic accuracy of PCR for TB, according to one meta-analysis [5], is not sufficient to diagnose ocular TB routinely, but a negative test may help avoid unnecessary prescription of long-term ATT. 

Multiple epidemiologic studies have established the relationship between TB and autoimmunity. Non-viable *M. tuberculosis* organisms can lead to immunologic responses, such as phlyctenular disease or Eales disease. The mechanism of such hypersensitivity reactions is poorly understood [11,12]. Unusual cases with overlapping features of TB and VKH reported in the literature may serve as preliminary evidence for a VKH-like hypersensitivity reaction in pre-existing TB [13]. Further investigation of a possible link between the development of VKH disease as a hypersensitivity reaction to IOTB should be undertaken using molecular and genetic techniques. 

## Conclusions

This case reveals an atypical manifestation of tuberculous uveitis mimicking VKH disease. Such cases highlight the importance of thorough investigation and timely diagnosis and treatment of TB and VKH diseases, whose managements differ substantially. In endemic regions, it is prudent to consider ocular TB even in the absence of pulmonary manifestations. Both entities entail potentially devastating visual and systemic consequences if not managed appropriately. 
